# President Obama’s Humble Face: An Authentic or a Socially Desirable Posturing? A Study on Reactions to Obama’s Autobiographical Self-Disclosures

**DOI:** 10.3389/fpsyg.2022.911556

**Published:** 2022-06-21

**Authors:** Alessia Mastropietro, Peter Bull, Francesca D’Errico, Isora Sessa, Stefano Migliorisi, Giovanna Leone

**Affiliations:** ^1^Department of Psychology, Sapienza University of Rome, Rome, Italy; ^2^Department of Psychology, University of York, York, United Kingdom; ^3^Department of Education, Psychology, and Communication, University of Bari “Aldo Moro”, Bari, Italy; ^4^Department of Communication and Social Research, Sapienza University of Rome, Rome, Italy

**Keywords:** humility, Obama, FACS, political speech, autobiographical social sharing, emotions, contempt

## Abstract

Referring to the mainstream studies based on the personalization’s hypothesis, which positively evaluates signals of dominance shown by leaders, the analysis of Obama’s rhetoric stays a relevant exception. His risky recall, during his political talks, of his social difficulties as a child of a mixed couple was in fact one of the more surprising aspects of his success. Nevertheless, reactions to his autobiographical sharing were scarcely explored. Based on the idea that these self-disclosures signal his responsivity toward the audience of low social condition and can, therefore, be defined as a sign of humility, this research aims to test if coherence between Obama’s words and his facial expressions of contempt, due to the seriousness of social injustices endured in his childhood, may influence the receivers’ perception of such unexpected communication. Before reading a brief autobiographical sharing taken from a “Back-to-school” speech, a highly ritualized monolog the US President addresses each year to students, 175 Italian participants were presented with a photo of Obama displaying either an expression of contempt (taken from the video of the speech) or a neutral expression. Comparisons between self-assessments of perceptions and reactions of participants assigned to the two experimental conditions show that a facial expression of contempt, coherent with words describing his school difficulties, has been crucial for perceiving this humble political discourse as authentic and not as a simple socially desirable posturing. More studies seem to be needed, however, to understand how humble speech could enhance the positive face of leaders or backfire against them.

## Introduction

This article is based on the idea that expressions of humility can positively contribute to politicians’ facework, meant to show a positive face to their audience (Bull and Fetzer, 2010), but only when specific situational and interactive conditions are fulfilled. In this study, we started to test this idea by first analyzing a genre of political communication that Bull and Fetzer (2010) defined as a “monolog.” In this specific situation, we assumed that the interactive condition leading to a good contribution of humility’s expressions to the politician’s positive face was the receivers’ perception that this humble communication was sincere. However, before discussing the hypotheses tested in the study and the choice of the monolog analyzed, a brief review of studies on humility in political communication is in order.

### Humility in Political Speech

Humility can be defined as a multimodal public stance interactively performed through verbal and non-verbal signals, whereby the person places themselves in a horizontal relationship to the interlocutor (D’Errico and Poggi, 2019). Therefore, a multimodal analysis seems to be the best methodological choice to observe and understand this kind of communication. In particular, the humble stance may be defined according to several aspects, namely, the tendency to express positive other-oriented emotions (e.g., empathy and compassion), the ability to regulate self-oriented emotions in socially acceptable ways (e.g., pride or excitement about one’s accomplishments; Davis et al., 2013), and the orientation of showing responsiveness to others’ needs (Fetzer and Bull, 2012). Considering recent studies on political personalization, a humble stance can seem useless, if not counteractive and incoherent, since it may be perceived as “unauthentic” (Luebke, 2020), especially in male politicians (Liu, 2016; D’Errico, 2019). Nevertheless, it could become very effective in conflictual situations, in which a humble stance could help speakers to better elaborate their own point of view.

Interestingly, a recent content analysis of common sense on humility (Weidman et al., 2018) highlighted a two-sided representation, which authors, respectively, named self-abasing humility and appreciative humility. The self-abasing humility, or modesty, motivates a speaker to hide from others and is associated with feelings of submissiveness, worthlessness, and traits, such as introversion and low self-esteem. Appreciative humility is instead associated with compassion, grace, and understanding, as well as with high status, high self-esteem, and agreeableness. The appreciative dimension of humility is related to behaviors, such as giving space to others’ opinions, admitting one’s own mistakes and gaps, and giving others’ merits (Liu, 2016; Weidman et al., 2018), as it seems to be based on a deep self-awareness of both one’s own strengths and limits (Tangney, 2000).

Based on such an appreciative definition of humility, recent studies explored the important role of emotional expressiveness in the perception of the politician’s authenticity.

On the one hand, overt expression of emotions, such as when showing anger or sadness, helps politicians to emphasize the importance of the topic at hand (Scardigno et al., 2021). However, when the specific case of humble communication is at stake, different pragmatic effects were observed depending on the specific emotion shown. Male political leaders proved to be especially effective when conveying a moral message and showing an angry facial expression; but they were perceived negatively, or their message was supposed to be hypocritical, when showing a sad expression (D’Errico, 2019). On the contrary, humble communication of female politicians seemed to elicit positive evaluations of their competence and benevolence when they exhibited a sad facial display during a persuasive message conveying a moral gist (D’Errico et al., 2022). This difference could be accounted for if considering the stereotypical expectancies linked to the social role assigned to men and women. While men are expected to be dominant, and therefore ready to express emotions more extroverted and linked to activity, such as rage, women are expected to express emotions linked to introversion and passivity, such as sadness (Eagly, 1987, 2005). Emotions shown by leaders could, therefore, be judged either as spontaneous or faked, depending on their coherence with previous expectations held by receivers. Similarly, referring to the social dominance of the group from which leaders originated, leaders of dominant groups are expected to be more agentic than the dominated ones.

Based on this line of thought, it is interesting to observe that one main rhetorical strategy used consistently by Barack Obama, both when speaking as an incumbent and when playing the official role of US President, was to overtly refer to him being the son of a mixed married couple, i.e., a social origin illegal in some North American states at the time of his birth. We speculated (Leone et al., 2015, 2018b) that, choosing such an authentic self-presentation (Gunn, 2015), he acted in a way that Arendt (1978) defined as being an “aware pariah,” i.e., an offspring of a seriously dominated group that shows pride and gratitude for his social origin instead that conceals it. According to the definitions of appreciative humility, we consider his unveiling of autobiographical memories of difficulties he encountered during his childhood and adolescence as a good instance of a humble stance. Moreover, we sustain that, when speaking to an audience of similar dominated social origins, this social sharing of autobiographical memories could also be taken as a sign of responsive humility.

### The Case Study

Within this theoretical framework, the case study selected refers to a “Back-to-school” speech, delivered by the former US President Barack Obama on 8 September 2009, in front of students of low socioeconomic status at the Wakefield High School of Arlington, Virginia. The “Back-to-school” Presidential address is a highly ritualized monolog, where each American leader in charge is expected to encourage students, at the beginning of every year’s lessons, to make their best to become competent citizens, aware that the future of their community depends on them. In a previous study (Leone et al., 2018a), we analyzed in-depth two Back-to-school speeches of Barack Obama, addressing students either of high or of low social status. Although in both speeches the President shared with students an autobiographical recall of serious difficulties of his adolescent times, a multimodal analysis highlighted that he expressed an emotion coherent with his verbal contents only in the speech addressed to the students of disadvantaged social status. In a frame-by-frame observation of this speech, in fact, we detected a facial expression of contempt, lasting less than a quarter of a second, coherent with his words of indignation for scholastic difficulties solely originated from disadvantaged social conditions that he declared to have personally known. This micro-expression (Ekman and Friesen, 1969), immediately regulated, revealed Obama’s emotion also beyond his communicative intentions. Interestingly, contempt like anger occurs after social or moral transgressions. Nevertheless, unlike anger, contempt only arises when social exclusion of targets is attributed to the unresponsiveness of actors (Fischer and Giner-Sorolla, 2016). Implying a social distancing as well as a self-regulatory function, contempt, therefore, coherently echoed both Obama’s severe words against marginalization of low-status students and the immediate regulation of his emotional expression. We decided, therefore, to observe if the presence or the absence of this contempt expression could affect the participants’ reactions to this humble Obama’s speech.

To answer this research question, we randomly assigned participants either to a condition in which the expression of contempt taken from the Back-to-school speech preceded the reading of the transcript of a part of Obama’s speech (experimental condition) or to a control condition in which participants observed a neutral expression of Obama’s face before reading the same transcript.

To build up the stimulus for the control condition, we first selected, from the same video, a still image of Obama’s face showing, according to FACS analysis (Ekman et al., 1978; Hager et al., 2002), no emotional expressions. We then asked participants to a pilot study to assess the expression shown in both images, choosing from a list of emotions, including “contempt” and “neutral.” The results showed that all participants recognized the expression of contempt. However, many participants attributed an emotional value also to the neutral image. We explained these wrong perceptions by the fact that, being caught from a video of Obama speaking, the image had a dynamic quality misleading participants’ perceptions. To help participants to recognize Obama’s face as a neutral one, we chose, therefore, a posed photo. However, being this photo a well-known one, questions were introduced to control the familiarity of Obama’s image and attitudes toward him.

Finally, to avoid the social desirability effects, participants were asked to describe their reactions to Obama’s speech following semi-projective instructions of putting themselves in the shoes firstly of a low-status student and then of a high-status student listening to this presidential address.

### Hypotheses

Based on the already discussed literature, we expect that participants reading President Obama’s self-disclosure of his own school difficulties, after viewing the image showing Obama’s contempt, i.e., a facial expression consistent with his words, will perceive his humble speech in more positive terms, evaluating it as more sincere. Instead, we expect that the inconsistency between the verbal content and the neutral face shown in the control condition will provoke an evaluation of falsehood and hypocrisy, leading to a negative perception of the speaker *(Hypothesis 1).* In addition, we expect a difference in the reactions of participants, depending on identification suggested in semi-projective instructions. Specifically, we expect that identifying with a low-status student will provoke more positive reactions *(Hypothesis 2)*, while identification with a student who does not experience socioeconomic difficulties will cause more negative reactions *(Hypothesis 3).* In this first explorative study, only aimed at observing effects on receivers of the facial expressions of the speaker, participants’ endorsement of gender stereotypes, although taken into consideration of introducing the issue of humble political speech, was neither measured nor controlled.

## Method

### Participants

By means of G*power (Faul et al., 2007), an *a priori* power analysis of the required sample (Lakens, 2022) for ANCOVA was performed with a power of 0.95, an α of 0.05, a medium effect size [*f* = 0.30, eta square: 0.08, using Cohen (1988) criteria], and one predictor (i.e., familiarity); this analysis indicated that a sample of 152 participants was required. Our sample’s size complied with this analysis, since 175 participants (122 women; 52 men; 1 other; average age of 22 years, DS = 4.84; 127 high school graduates and 48 graduates) were recruited as a snow-ball convenience sample and randomly assigned either to the control (*N* = 95) or to the experimental condition (*N* = 80). The study was conducted during COVID-19 restrictions due to online participation. Despite its limitations, we judged the benefit of this first collection of explorative data worthwhile.

### Stimuli

**FIGURE 1:**
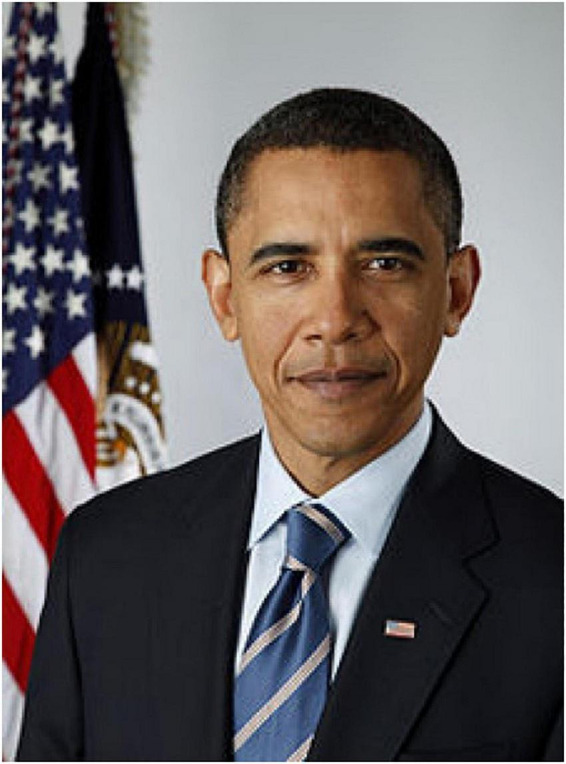
Neutral facial expression accompanying the speech excerpt in the control condition. Image licensed under Creative Common Attribution 3.0 License. Photographed and attributed to Pete Souza. Available via https://it.wikinews.org/wiki/File:Official_portrait_of_Barack_Obama.jpg.

**FIGURE 2:**
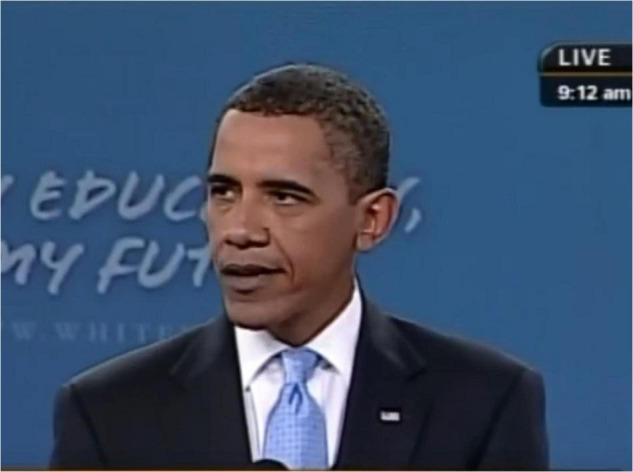
Facial expression of contempt accompanying the speech excerpt in the experimental condition. Screenshot taken from https://obamawhitehouse.archives.gov/copyright. Copyright policy details that pursuant to federal law, government-produced materials appearing on this site are not copyright protected. The United States Government may receive and hold copyrights transferred to it by assignment, bequest, or otherwise. Except where otherwise noted, third-party content on this site is licensed under a Creative Commons Attribution 3.0 License. Visitors to this website agree to grant a non-exclusive, irrevocable, royalty-free license to the rest of the world for their submissions to Whitehouse.gov under the Creative Commons Attribution 3.0 License.

As briefly discussed above, the coherence vs. incoherence between facial expression and words was induced using two images of Obama. In the lack of coherence condition (control condition), a well-known photo of Obama’s face showing no emotional expression was presented (Figure 1). In the coherence condition (experimental condition), we used a still image taken from the video of the “Back-to-school” speech at Wakefield High School (Figure 2). Having observed this video through a FACS analysis (Ekman et al., 1978; Hager et al., 2002) in a previous descriptive study (Leone et al., 2018a), a micro-expression of contempt (Left Unilateral Action Unit 14) appeared on his face for less than a quarter of a second when Obama said: *“I get it. I know what that’s like*.”

### The Transcript of the Speech at Wakefield High School

As already discussed, the excerpt chosen as an instance of Obama’s humble stance refers to his autobiographical social sharing and runs as follows (you can find the complete video-recording of this “Back-to-school” speech at,^1^ being this excerpt running from 9.12 to 9.48 min):

«*I know it’s not always easy to do well in school. I know a lot of you have challenges in your lives right now that can make it hard to focus on your schoolwork. I get it. I know what it is like. [.] I was raised by a single mother who struggled at times to pay the bills and wasn’t always able to give us things the other kids had. There were times when I missed having a father in my life. I was lonely and felt like I didn’t fit in.*

*I did some things I’m not proud of and got in more trouble than I should have. And my life could have taken a turn for the worse. But I was lucky*».

### Measures of Dependent Variables

#### Perception of the Speaker

After reading the text, participants were asked the question: *”Barack Obama’s words you just read, how do they look to you?”* To answer the question, participants used a list of 26 adjectives, evaluating each one of them on a scale ranging from 0 (not at all) to 3 (a lot). The adjectives included in the list were either positive (“*affectionate,” “authentic,” “warm,” “understanding,” “convincing,” “empathetic,” “encouraging,” “participatory,” “reassuring,” “sincere,” “spontaneous,” “touching,” and “close”)* or negative *(“artifact,” “exaggerated,” “false,” “out of place,” “cunning,” “hypocritical,” “manipulative,” “paternalistic,” “rhetorical,” “honeyed,” “strategic,” “cold,” and “weak”)*. Adjectives were randomly presented to avoid the order effect.

#### Emotional Reactions to the Speech

Participants were then asked to put themselves in the shoes of a student in a condition of socioeconomic difficulty listening to Barack Obama’s speech and to indicate their reactions to it. Immediately, the same question was proposed, asking participants to identify with a student in medium-high socioeconomic condition. To answer both questions, a list of 32 reactions was presented, asking participants to assess their degree of agreement for each item from 0 (not at all) to 3 (a lot). The reactions were either positive (“*welcomed,” “involved,” “impressed,” “encouraged,” “proud,” “reassured,” “surprised,” “inspired,” “moved,” “empowered,” “stimulated,” “valued,” “understood,” and “excited”)* or negative *(“uncomfortable,” “embittered,” “bored,” “angry,” “would have felt contempt,” “would have felt shame,” “disappointed,” “embarrassed,” “afraid”, “guilty,” “indifferent,” “indignant,” “irritated,” “perplexed,” “sad*,*” and “offended”)* and were randomly presented.

#### Control Measures

To check familiarity, we asked participants to answer on a scale from 0 (not at all) to 3 (very) to the question “*How familiar is Obama to you?”*. To control the opinions held on Obama, we asked participants to answer the question “*Do you think Obama got his position*,” choosing among the prearranged answers: *by merit, fortunately, by strategy, or something else*. To check the degree of sympathy toward Obama, we proposed to the participants to evaluate a semantic differential on a 5-step scale from 0 (“*dislike*”) to 5 (“*sympathy*”) by answering the question *“What do you feel when thinking of Obama?”* Finally, we asked for some personal information, including their political orientation to which participants could respond by positioning themselves in one of the prearranged answers: *Left*, *Center-left*, *Center*, *Center-right*, *Right*, or *something else*.

## Procedure

The experimental procedure was carried out through an online questionnaire, created with Google Forms. It allowed us to divide the questionnaire into several pages, so that the participants could see only one page at a time and cannot go back to previously filled-in pages. Before accessing the questionnaire, participants read the informed consent form and were asked to consent to the processing of data. Subsequently, participants were presented with a first text explaining that every year the President of the United States addresses students with a “Back-to-School” speech and that they will read an excerpt from one of these speeches, delivered by the US President Barack Obama. On the next page, participants were randomly assigned either to the group presented with Obama’s photo showing a neutral expression or to the group presented with Obama’s image showing an expression of contempt and were free to decide when moving to the next page, where they were presented with the excerpt of Obama’s speech. After taking their time for reading the excerpt, participants answered the questions investigating the dependent and control variables. Finally, they gave some information about themselves to better describe the sample.

## Results

To examine potential differences between experimental and control conditions (expression of contempt vs. neutral expression) concerning the dependent variables, we run a MANCOVA analysis for each adjective describing both Obama’s perception and participants’ emotional reactions. To reduce the type I error, we decided to set the significance level at p ≤ 0.025.

### Perception of Obama’s Self-Disclosure

The analysis of variance for the adjectives describing the speaker showed the following level of significance, also by taking into account the Obama’s familiarity as a covariate: *Sincere* [*F*_(1,175)_ = 8.56, *p* = 0.004, η2 = 0.047], *Spontaneous* [*F*_(1,175)_ = 6.74, *p* = 0.010, η2 = 0.038], *Touching* [*F*_(1,175)_ = 5.47, *p* = 0.020, η2 = 0.031], *Artifact* [*F*_(1,175)_ = 8.71, *p* = 0.004, η2 = 0.048], *Exaggerated* [*F*_(1,175)_ = 10.59, *p* = 0.001, η2 = 0.058], *False* [*F*_(1,175)_ = 9.91, *p* = 0.002, η2 = 0.054], *Cunning* [*F*_(1,175)_ = 10.37, *p* = 0.002, η2 = 0.057], *Hypocrite* [*F*_(1,175)_ = 8.53, *p* = 0.004, η2 = 0.047], *Rhetoric* [*F*_(1,175)_ = 5.75, *p* = 0.018, η2 = 0.032], *Strategic* [*F*_(1,175)_ = 12.21, *p* = 0.001, η2 = 0.066], and *Weak* [*F*_(1,175)_ = 5.76, *p* = 0.017, η2 = 0.032]. Hence, as shown in Table 1, Obama’s discourse is perceived as more sincere, more spontaneous, and more touching when it is matched with the image of his expression of contempt. In the control condition, his communication is perceived instead as more artifact, more exaggerated, more false, more cunning, more hypocrite, more rhetoric, and more strategic, but also weaker.

**TABLE 1:** Average scores and standard deviation of perception’s adjectives in the experimental and control conditions (expression of contempt vs. neutral expression).

Perception’s adjectives	Expression of contempt		Neutral expression	
		
	*M*	DS	*M*	DS
Sincere	2.58	0.63	2.24	0.82
Spontaneous	2.09	0.90	1.73	0.90
Touching	2.09	0.83	1.78	0.88
Artifact	0.25	0.46	0.55	0.78
Exaggerated	0.14	0.35	0.41	0.68
False	0.04	0.19	0.27	0.64
Cunning	0.40	0.70	0.81	0.93
Hypocrite	0.09	0.28	0.34	0.71
Rhetoric	0.56	0.74	0.88	0.97
Strategic	0.80	0.83	1.29	0.99
Weak	0.18	0.47	0.39	0.66

### Semi-Projective Self-Assessment of Participants’ Reactions

Participants were asked to self-assess their reactions to Obama’s autobiographical recall trying to identify themselves firstly with a student in a difficult socioeconomic condition and then with a student in a medium-high economic condition.

Applying a MANCOVA analysis, with Obama’s familiarity as a covariate, to self-assessments collected according to the first suggestion (identify with a student of low socioeconomic condition), depending on their experimental conditions, participants differently self-assessed themselves in reference to the adjectives *Interested* [*F*_(1,175)_ = 6.08, *p* = 0.015, η2 = 0.034], *Outraged* [*F*_(1,175)_ = 5.25, *p* = 0.023, η2 = 0.030], *Perplexed* [*F*_(1,175)_ = 10.26, *p* = 0.002, η2 = 0.056], and *Offended* [*F*_(1,175)_ = 6.55, *p* = 0.011, η2 = 0.037]. More precisely, participants in the experimental condition declared to be more interested in reading Obama’s autobiographical self-disclosure than participants presented with an image showing a neutral expression. Contrariwise, participants declared to be more outraged, more perplexed, and more offended when reading Obama’s words after seeing the photo showing a neutral expression (Table 2).

**TABLE 2:** Comparison of significantly different medium scores and standard deviation of participants identifying with students in low economic conditions, according to experimental and control conditions (expression of contempt vs. neutral expression).

Reactions	Expression of contempt		Neutral expression	
		
	*M*	DS	*M*	DS
Interested	2.39	0.70	2.11	0.78
Outraged	0.06	0.24	0.21	0.52
Perplexed	0.40	0.56	0.74	0.77
Offended	0.06	0.24	0.21	0.46

Interestingly, participants identifying themselves with a student in a medium-high economic condition, when compared between the experimental and the control condition, did not show any significant difference.

## Discussion

Taken all together, the results seem to confirm our first hypothesis. The perception of the same autobiographical social sharing, in fact, deeply varied according to the information conveyed by Obama’s facial expression, shown before the reading. We could articulate that coherence between words and facial emotional expression may account for the humble utterance to be sincere, significantly reducing the social distance between this prominent political leader and his audience. On the contrary, the incoherence between his touching words and his neutral face shown before reading could lead perceivers to consider the humble attitude of Obama only as a socially desirable, if not hypocritical, posture. An interesting facet of this data refers to the adjective “weak.” Data suggest that a humble speaker appears weak to his audience not when expressing a controversial reaction, such as contempt, but only when his face is not leaking the same indignant attitude conveyed by his words. This could help to better understand why, in previous studies on humble leaders, we found that they were negatively perceived when discussing moral topics (D’Errico, 2019).

Regarding the second hypothesis, only some of our theoretical expectations were proved true. When participants put themselves in the shoes of students of low social condition, they were interested in this unusual communicative move, only when Obama’s facial expression was coherent with his words. However, if such passionate and unusual words were associated with a neutral expression, then they backfired, provoking negative reactions instead of social closeness. Interestingly, the falling short of expectancies related to reactions of participants identifying with high-status students suggests that the humble self-exposure of Obama was only fine-tuned for students of low social status: precisely the kind of audience he was addressing during his “Back-to-school” speech. To summarize, the importance of this interconnection between bodily communicative signals, relational humble stance, and potential features of the audience (low or high socioeconomic condition) shed the first light on the complexities of the persuasiveness of the humble political speech. Future studies should better consider other participants’ characteristics, such as political engagement and attitude, or individual differences in the social dominance’s orientation.

## Conclusive Remarks

Based on the idea that a humble stance during political speeches may either enhance the positive face of political leaders or backfire against them, this study aimed to explore the consequences of Obama’s humble sharing of his school difficulties with students of low social condition, listening to a “Back-to-school” discourse he delivered when he was the US President. In the framework of a multimodal analysis of communication, the Independent Variable manipulated was the coherence vs. the lack of coherence between two facial expressions of Obama (contempt vs. neutral), shown before presenting participants with an excerpt of the talk conveying a brief autobiographical sharing of his school difficulties. Results highlight how much participants’ reactions changed, only because of seeing Obama’s facial expression of contempt. It could be argued that for receivers, the leaking of a powerful speaker’s emotions during an official talk can act as an indirect yet powerful signal of authenticity, especially when an unexpected self-exposure puts a powerful politician to the same level of his audience. However, this is only a first explorative study, presenting some important limitations, and many questions remain unsolved.

The first limitation is represented by the different kinds of images used for our manipulation. In fact, to prevent the distortions of Obama’s neutral expression observed in the pilot study, the experimental condition showed a still image taken from Obama’s speech, while the control condition showed a posed photo. Moreover, in the original Back-to-school speech, contempt appeared as a micro-expression. Since our procedure aimed at the first exploration of the effects of congruence between facial expressions and verbal contents of a humble political speech, we decided to show this micro-expression as a still image to be observed for several seconds, seriously changing the features of the original case study. Nonetheless, since this first study shows how an emotional expression congruent with words influences participants’ perceptions and reactions to a politician’s humble speech, we can now design further studies based on new and more sophisticated procedures. The effects of micro-expressions could now be studied as an I.V. *per se*, by importing procedures developed for this field of study (cf. Stewart et al., 2009). For instance, participants could be presented either with the original video, showing the micro-expression, or with a second video, manipulated, where the micro-expression is replaced by a neutral expression, to assure the two videos have the same length. Moreover, the sample size of participants, although complying with an *a priori* power analysis settled at the classic 0.05 level of significance, was slightly under the number requested for the more prudent level of significance applied. Finally, our study did not explore the participants’ gender stereotypes, although the literature on humble political speech proved this aspect to be relevant in shaping the participants’ expectancies about political speakers. Further studies may change the research’s design, making this variable interact both with the multimodal communicative congruence and with different kinds of emotions shown by the speaker.

Many other research questions may be addressed. The effects described in this study could be partially due to the special intimacy produced by the social sharing of autobiographical memories, and other kinds of humble utterances could lead to different reactions. In addition, specificities of contempt must be better explored in comparison with cognate emotions (i.e., rage, sadness). Finally, the effects of humble communication in political speeches different from “monologs” (Bull and Fetzer, 2010) must be analyzed. In fact, humility’s consequences could deeply vary, according to the symmetric or asymmetric relations between speakers and audiences. The categorization of political speeches according to a decreasing symmetry (ranging from the highest asymmetry of monologs to the more balanced relationship between politicians and journalists and to the symmetry of Question Times and Parliamentary debates) (Bull and Fetzer, 2010) offers a useful grid to further develop the first ideas presented in this study.

## Data Availability Statement

The raw data supporting the conclusions of this article will be made available by the authors, without undue reservation.

## Ethics Statement

The studies involving human participants were reviewed and approved by the Ethical Committee of the University of Bari. All the procedures followed the Helsinki ethical principles and ethical codes of AIP (Italian Psychology Association). Informed consent was obtained for all participants. The patients/participants provided their written informed consent to participate in this study.

## Author Contributions

AM and GL designed the study. IS and SM realized the procedure and collected the data. All authors analyzed the results and co-wrote the article.

## Conflict of Interest

The authors declare that the research was conducted in the absence of any commercial or financial relationships that could be construed as a potential conflict of interest.

## Publisher’s Note

All claims expressed in this article are solely those of the authors and do not necessarily represent those of their affiliated organizations, or those of the publisher, the editors and the reviewers. Any product that may be evaluated in this article, or claim that may be made by its manufacturer, is not guaranteed or endorsed by the publisher.

## Footnotes

1
https://www.youtube.com/watch?v=8ZZ6GrzWkw0
